# Application of an In Vitro Digestion Model for Wheat and Red Beetroot Bread to Assess the Bioaccessibility of Aflatoxin B_1_, Ochratoxin A and Zearalenone and Betalains

**DOI:** 10.3390/toxins14080540

**Published:** 2022-08-08

**Authors:** Paula Llorens Castelló, Ana Juan-García, Juan Carlos Moltó Cortés, Jordi Mañes Vinuesa, Cristina Juan García

**Affiliations:** Laboratory of Food Chemistry and Toxicology, Faculty of Pharmacy, University of Valencia, 46100 Burjassot, Spain

**Keywords:** bioaccessibility, aflatoxin B1, ochratoxin A, zearalenone, bread, beetroot, LC-Q-TOF-MS

## Abstract

Nowadays, the bakery industry includes different bioactive ingredients to enrich the nutritional properties of its products, such as betalains from red beetroot (*Beta vulgaris)*. However, cereal products are considered a major route of exposure to many mycotoxins, both individually and in combination, due to their daily consumption, if the cereals used contain these toxins. Only the fraction of the contaminant that is released from the food is bioaccessible and bioavailable to produce toxic effects. Foods with bioactive compounds vary widely in chemical structure and function, and some studies have demonstrated their protective effects against toxics. In this study the bioaccessibility and bioavailability of three legislated mycotoxins (AFB_1_, OTA and ZEN), individual and combined, in two breads, one with wheat flour and the other with wheat flour enriched with 20% *Beta vulgaris*, were evaluated. Bioaccessibility of these three mycotoxins from wheat bread and red beet bread enriched individually at 100 ng/g was similar between the breads: 16% and 14% for AFB1, 16% and 17% for OTA and 26% and 22% for ZEN, respectively. Whereas, when mycotoxins were co-present these values varied with a decreasing tendency: 9% and 15% for AFB1, 13% and 9% for OTA, 4% and 25% for ZEN in wheat bread and in red beet bread, respectively. These values reveal that the presence of other components and the co-presence of mycotoxins can affect the final bioavailability; however, it is necessary to assess this process with in vivo studies to complete the studies.

## 1. Introduction

Cereals and cereal-based products play a crucial role in the human diet and livestock feed, due to their valuable nutritional content, such as carbohydrates, proteins, fatty acids and vitamins [1]. However, these characteristics make cereals a good source of nutrients for the growth and appearance of fungi. In the case of mycotoxigenic fungi, it represents a toxicological risk that has to be controlled and, as far as possible, prevented. Mycotoxigenic fungi stand out for their ability to grow in a wide spectrum of climatic conditions. Fungal contamination of crops can be carried out at all stages of production; pre-harvest and harvest (in the field) or in the post-harvest stages (transport and storage). Good handling practices and a hazard analysis and critical control point (HACCP) system are essential in prevention of fungal contamination. However, in many cases, such systems are not applied and the presence of mycotoxins becomes possible. *Aspergillus* and *Fusarium* species colonize cereals and cereal products, and they are producers of the following three most toxicological mycotoxins: aflatoxins (*Aspergillus flavus* and *Aspergillus parasiticus*), ochratoxin-A (OTA) (*Aspergillus ochraceus* and *Penicillum verucosum*) and zearalenone (ZEN) (*Fusarium graminearum*, *F. culmorum*, *F. equiseti*, and *F. verticillioides*). The intake of these mycotoxins could cause health effects, such as mutagenicity, dermato-toxicity, neurotoxicity, hepatotoxicity, teratogenicity, estrogenicity, carcinogenicity and immunosuppressive effects [2,3,4,5]. Furthermore, the IARC has classified aflatoxins (AFs) as agents of carcinogenicity. OTA is possibly carcinogenic to humans and ZEN is not classifiable as regards humans. Due to these effects in humans and animals, national and European legislative institutions have established maximum tolerable levels of AFB1 and OTA in bakery products to protect consumers from the health risks associated with their intake. It is established in EC Regulation No. 1881/2006 [6] that the highest legislative content for products derived from cereals, such as bread, is 2 μg/kg for AFB1, 3 μg/kg for OTA and 100 or 50 μg/kg for ZEN in cereal products or bread, respectively [6].

After ingestion of food contaminated by AFB1, OTA or ZEN, the quantities of mycotoxins that are absorbed gastro-intestinally and reach the circulation system determines their toxicological effect. In this sense, bioaccessibility is necessary to elucidate the risk assessment. Furthermore, previous studies have elucidated that the presence of other compounds, or the dietary source, influences the percentage of bioaccessibility. In wine, OTA presented 26% bioaccessibility [7]. Versantvoort et al., [8] evaluated the effect of a food-mix of peanut slurry, buckwheat and standard meal on the bioaccessibility of aflatoxin B1 (AFB1) and OTA, obtaining 84% for AFB1 and 86% for OTA in combined contamination, and 83% for AFB_1_ and 100% for OTA in single contamination.

Red beetroot is rich in bioactive ingredients that ensure health-promoting effects and it is recognized as a functional food, due to its high nutritional value. Red beet is commonly consumed and used for manufacturing food coloring agents. Its consumption has witnessed a pronounced increase. In bakery, it is used to increase nutritional value of bakery products and also to obtain colorimetrically acceptable and tasty products. Furthermore, red beet is the precursor of betalains that have become popular because they have potent antioxidant, anticarcinogenic, hepatoprotective, antibacterial and anti-inflammatory activities, as well as intestinal and immune regulatory effects. In addition, they protect cells against peroxidation and DNA damage [9]. Recently, Penalva-Olcina et al., [10] observed the effective protection of beetroot extract in SH-SY5Y neuronal cells against Fumonisin B1, Ochratoxin A and their combination. Beetroot extract in bread or biscuits, as a product of daily consumption, can increase its intake and health effects.

Due to the health-related benefits, and as organoleptic ingredients, it would be a good protective strategy to attenuate the biological effects of AFB_1_, OTA and ZEN in case they are ingested. However, to consider these effects it is first necessary to evaluate the bioaccessibility of both types of compounds. In this study, the bioaccessibility of three different legislated mycotoxins (AFs, OTA and ZEA), individually and combined, in two breads, one with wheat flour and the other with wheat flour enriched with *Beta vulgaris*, was evaluated. Furthermore, the bioaccessibility of some betalains, identified in enriched bread, all through the different digestive phases (salival, gastric and intestinal) were studied.

## 2. Results and Discussion

### 2.1. Fortification Levels of AFB_1_, OTA and ZEN Analysis

European legislation has established maximum levels (MLs) for OTA, AFB_1_ and ZEN in all products derived from unprocessed cereals, including processed cereal products and cereals intended for direct human consumption. Such levels are set at 3 and 2 μg/kg for OTA and AFB_1_, respectively [6]. For unprocessed cereals the maximum level is set at 5 μg/kg for OTA. However, in processed cereal-based foods and baby foods for infants and young children the maximum is set at 0.5 and 0.1 μg/kg for OTA and AFB_1_, respectively [6]. The highest values of ML for ZEN have been established as follows: 100 μg/kg for unprocessed cereals; 50 μg/kg in bread, including small bakery wares, pastries, biscuits, cereal snacks and breakfast cereals, excluding maize snacks and maize-based breakfast cereals; and 20 μg/kg for processed cereal-based foods (excluding processed maize-based foods) and baby foods for infants and young children. Despite these regulations and food controls, the mycotoxins have been detected in routine customs analysis and in products from various countries; in some cases, in bread from Guyana, Morocco, Portugal, Turkey and Malaysia, at levels 30 times above the maximum limits (MLs) [11,12,13,14,15,16]. Surprisingly, it is possible that many consumers have ingested these mycotoxins through one of the most consumed food products, bread.

In literature, different studies indicate that during the bakery process a reduction in the presence of mycotoxins by 7–85% and 6–40% for OTA and AFB_1_, respectively, has been observed [17,18]. For this reason, this study was performed after the preparation of the bread and it was established that the studied levels of fortifications would be at two levels, 100 μg/kg and 10 μg/kg, before the digestion process, by adding 1 mL of the working solutions (100 µg/mL) in 10 g of wheat bread.

### 2.2. Bioaccessibility of OTA, AFB1 and ZEN in Studied Breads

Salival, gastric and intestinal phase digestions were evaluated. However, the bioaccessibility was calculated at the last phase, corresponding to the intestinal phase. These data were analyzed for both beet and wheat bread, comparing the differences in bioaccessibility between the levels of single mycotoxins and their combinations. The bioaccessibility value obtained was a percentage value, representing availability to be absorbed at the intestinal level, and it assumed the free fraction of the matrix. Therefore, the most relevant bioaccessibility results were those corresponding to the intestinal phase. Although, it should not be forgotten that a slight absorption of the components can occur throughout the gastrointestinal tract. For this reason, data from the other phases were collected and are discussed in this work.

Availability was calculated for all three phases, and it was calculated as the percentage of mycotoxins from the bread that was detected in extracts from each phase, using the formula: Availability = [(CDE)/(CB)] ∗ 100. (CDE: concentration in digested extract and CB: concentration in bread. 

#### 2.2.1. Availability of AFB1, OTA and ZEN in Salival, Gastric and Intestinal Phase in Both Breads

**Figure 1:**
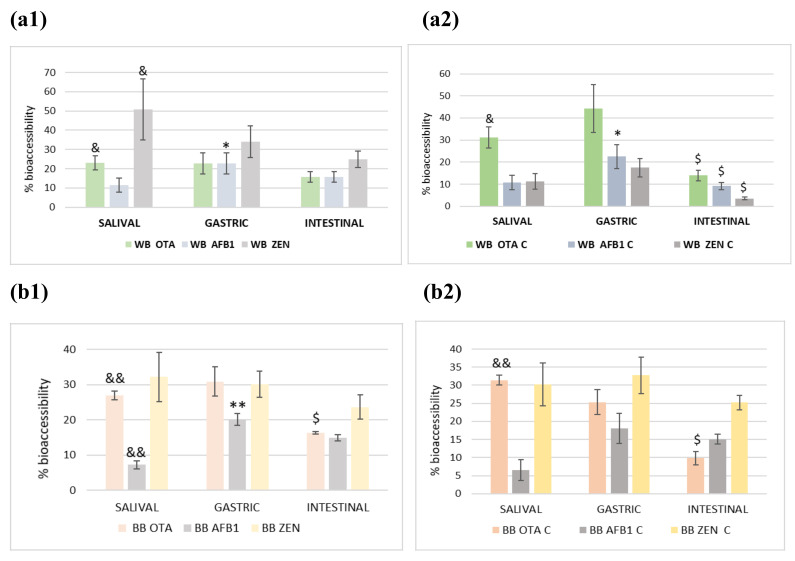
Percentage of digested mycotoxin (%) in three studied phases of: (**a**) wheat bread at 100 ng/g individual (**a1**) and combined fortification (**a2**); (**b**) beet bread at 100 ng/g individual (**b1**) and combined fortification (**b2**) (*: Salival-Gastric *** *p* < 0.001; ** *p* < 0.01; * *p* < 0.05; $: Gastric-Intestinal $$$ *p* < 0.001; $$ *p* < 0.01; $ *p* < 0.05; and &: Salival-Intestinal &&& *p* < 0.001; && *p* < 0.01; & *p* < 0.05).

The percentage of ZEN progressively decreased during the digestion process at a fortification level of 100 ng/g in individual (51% to 22%) and combined (10% to 5%) presence in wheat (Figure 1(a1)). In red beet bread (Figure 1(b1)) a decrease in values in the intestinal phase was observed, but all values were above 23%. Regarding OTA and AFB1, values presented variably along the digestion phases, reaching 15% in the intestinal phase in wheat bread for both mycotoxins (Figure 1(a1)), and 17% and 15% in red beet bread (Figure 1(b1)), respectively. OTA in the gastric phase presented higher percentages (20%, 42%, 31% and 25%) than in the saliva and intestinal phases, probably due to the fact that the bread was less digested, because in this phase amylase and pepsin activities are present. However, in the intestinal phase pancreatin and bile are active, so more components were loose in the bolus and OTA was degraded.

According to the results obtained, availability in each phase increased significantly, at the low level of fortification (10 ng/g), reaching the intestinal phase with values up to 65% for OTA and ZEA and, in the case of AFB_1_, values of availability between 10–45% in wheat bread and slightly higher in beetroot bread with 25–45%. This fact might be due to adhesion mechanisms that favor its protection against the enzymes responsible for digestion. Contamination with these mycotoxins at low levels allows them to remain embedded in the food matrix, and, therefore, the percentage digested is lower, compared to those samples fortified at higher levels, where the mycotoxins are exposed to enzymatic action. Furthermore, the chemical structure is different, with AFB_1_ presenting a higher amount of lactone and pentanone than OTA or ZEN. Based on the results obtained, ZEN is highly bioaccessible (100%) in wheat bread when fortified at 10 ng/g.

Comparing both breads it was observed that red beet bread presented high availability in the three phases in both fortifications (single Figure 1(b1) and combined Figure 1(b2)). However, AFB1 presented the lowest values (8–16% and 6–15%). No differences were observed between individual or combined fortifications.

The highest statistically significant differences for all three mycotoxins (*p* < 0.05) were observed in combined fortifications between gastric to intestinal phases in wheat flour bread (Figure 1(a2) and Figure 1(b2)).

#### 2.2.2. Bioaccessibility of AFB1, OTA and ZEN in Both Breads

Bioaccessibility was calculated as the percentage of mycotoxins from the bread that were detected in intestinal phase extracts by the formula: Bioaccesibility = [(CDE)/(CB)] ∗ 100 in which CDE is the concentration in digested extract and CB is the concentration in bread.

**Figure 2:**
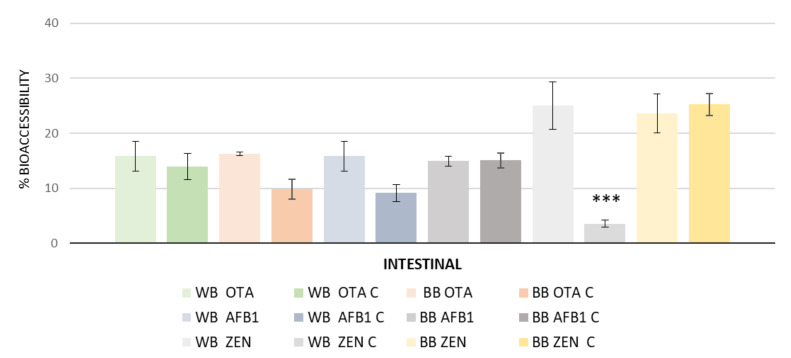
Individual (WB OTA, WB AFB_1_, WB ZEN) and combined (WB OTAC, WB AFB_1_C, WB ZENC) bioaccessibility results in wheat bread (WB) versus beet bread (BB) at 100 ng/g of mycotoxin (OTA, AFB_1_, ZEN) (*** *p* < 0.001; ** *p* < 0.01; * *p* < 0.05).

The results were analyzed as individual and combined (AFB_1_+OTA+ZEN) presence of mycotoxins in bread (Figure 2). The results showed that ZEN had high bioaccessibility in wheat flour bread as a single fortification (26%) and in red beetroot bread with both fortifications (22% for single and 26% combined with the other two mycotoxins). OTA presented values of 16% and 17% in wheat and beet bread, respectively, when fortified individually with 100 ng/g of OTA, and in combination AFB_1_ + ZEN at 100 ng/g values of 14% in wheat bread and 10% in red beet bread.

Similar values were observed with AFB_1_. However, in wheat flour bread combined with OTA+ZEN at 100 ng/g the bioaccessiblity was 9%, which was 8 units lower than wheat flour bread contaminated only with AFB1. The bioaccessibility of the three mycotoxins in bread were lower than those observed by other authors individually without meal. Sobral et al. [19] observed that AFB_1_ and OTA were released along GI digestion, exhibiting a maximum cumulative isolated bioaccessibility of 51.6 and 72.4%, respectively, after intestinal digestion.

Regarding interactions, the release of AFB1 or OTA was significantly affected by the presence of the other mycotoxin.

#### 2.2.3. Bioaccessibility of Betalains in Both Breads

**Figure 3:**
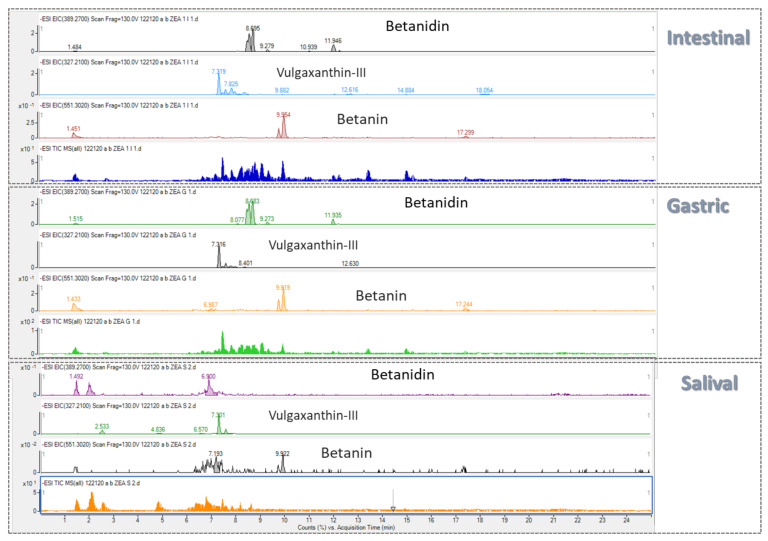
Total ion chromatograms (TIC) and extracted ion chromatograms (EIC) of betanidin, vulgaxanthin−III and betanin, from red beet bread digested phases (salival, gastric, intestinal).

Betanin is the only standard commercially available betalain and, therefore, it was the only compound of the betalain family of on which to perform further specific studies, such as qualitative and quantitative analyses. A tentative identification of betalains in dried beetroot powder was possible by LC-Q-TOF-MS which permitted the identification of seven betalains, two betacianins (betanin, betanidin) and four betaxanthins (Vulgaxanthin-III: Asparagine-betaxanthin; Vulgaxanthin-I: Glutamine-betaxanthin; Vulgaxanthin-II: Glutamic acid-betaxanthin; Tyrosine-betaxanthin). However, it was only possible to detect three betalains (betanin, betanidin and Vulgaxanthin-III) in the three phases of digestion and they were evaluated in digested red beetroot baked bread (Figure 3). In the literature, different studies indicate the total betalain content in red beet and derivative products [19,20]. Most recently, Sawicki et al., [21] studied thirteen varieties of red beets and reported betaxanthin levels ranging from 2.71 to 4.25 mg/g DW, whereas the content of betacyanins varied between 8.3–13.5 mg/g dry weight (DW).

**Figure 4:**
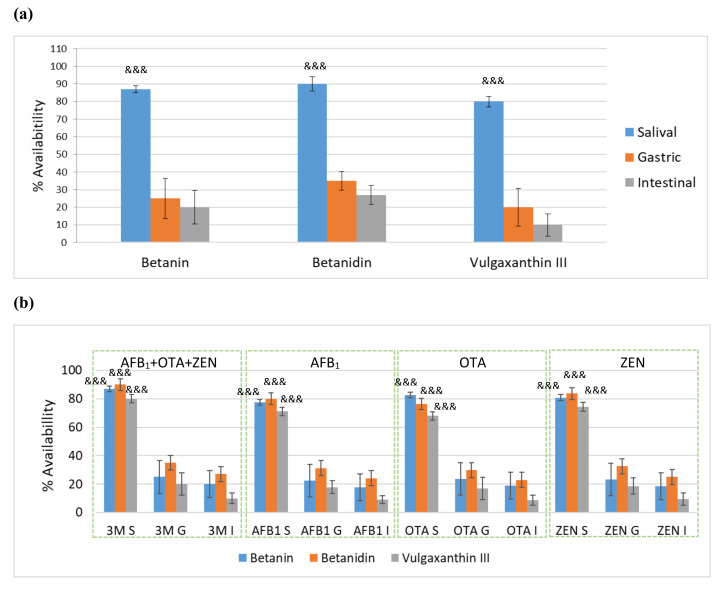
(**a**) Availability of three betalains (betanin, betanidin and vulgaxanthin-III) in beet-red bread. (**b**) Percentage (%) of three betalains (betanin, betanidin and vulgaxanthin-III) in three digested phases (S, G, I) of beet red bread contaminated with single (AFB_1_ S, AFB_1_ G, AFB_1_ I, OTA S, OTA G, OTA I, ZEN S, ZEN G, ZEN I) and combined (3M S, 3M G, 3M I) mycotoxins at 100 μg/kg. (*: Salival-Gastric *** *p* < 0.001; ** *p* < 0.01; * *p* < 0.05; $: Gastric-Intestinal $$$ *p* < 0.001; $$ *p* < 0.01; $ *p* < 0.05; and &: Salival-Intestinal &&& *p* < 0.001; && *p* < 0.01; & *p* < 0.05).

Regarding availability, the three betalain compounds mentioned above presented high concentrations (80–90%) in the salival phase (Figure 4a), but during gastric and intestinal digestion the quantities decreased and the percentage in the intestinal phase in beet bread with a combination of AFB_1_+OTA+ZEN (100 μg/kg) remained at 20%, 27% and 10% for betanin, betanidin and vulgaxanthin-III, respectively (Figure 4). Similar values were observed by Sawicki et al. (2020) for the gastric content after 2h of an intragastric administration of fermented red beetroot juice in rats for betanin and betanidin, obtaining values of 18% and 25%, respectively. These compounds in our study remained in intestinal bolus after digestion, so these amounts would be available to be absorbed in the intestinal tract. These results confirmed those shown by Sawicki et al. [22] in plasma and urine from healthy volunteers that consumed juice with a dose of 0.7 mg betalains/kg body weight, reaching the highest concentration in blood plasma (87.65 ± 15.71 nmol/L) and urine (1.14 ± 0.12 μmol) after the first and second weeks of juice intake. The study also indicated the presence of native betalains and their deglucosylated, decarboxylated, and dehydrogenated metabolites in human physiological fluids.

## 3. Conclusions

According to the results reported, the quantities of the studied mycotoxins and betalains decreased all along the digested phases to the intestinal space. However, a high bioaccessibility in red beetroot breads, without significant differences in the values obtained, was observed. Whereas, if the mycotoxins were combined, the bioaccessibility decreased, being more significant for ZEN with values of 4%. Betalains presented similar bioaccessibility if the mycotoxins were in single or combined presence. It was observed that their availability decreased during the different phases of digestion and had bioaccessibility values of 20%, 27% and 10% for betanin, betanidin and vulgaxanthin-III, respectively.

These values revealed that the presence of other components, and mainly the copresence of mycotoxins, could affect the final bioavailability. However, it is necessary to assess this complete process with in vivo studies to fully understand the entire progression.

## 4. Materials and Methods

### 4.1. Chemicals, Reagents and Equipment

Standard ochratoxin A (OTA), aflatoxin B_1_ (AFB_1_) and zearalenone (ZEA) were purchased from Sigma-Aldrich (St. Louis, MO, USA). Individual stock solutions of mycotoxins were prepared in AcN at 500 µg/mL and formed solutions in MeOH at 100 µg/mL. The solutions were maintained at −20 °C in the dark. Betanin was purchased from Sigma-Aldrich and diluted to prepare solution in EtOH.

To prepare the gastrointestinal solutions, KCl 89.6 g/L, KSCN 20 g/L, NaH_2_PO_4_ 88.8 g/L, NaSO_4_ 57 g/L, NaCl 175.3 g/L, NaHCO_3_ 84.7 g/L, urea 20 g/L and Milli-Q water (Milli-Q water purification system (Millipore, Bedford, MA, USA)) were used. First, 145 mg α-amylase in 100 mL of Milli-Q water was prepared. A pepsin solution with 0.5 mg pepsin (1 g in 25 mL HCl (0.1 N) (Pepsin from porcine gastric mucosa, powder, ≥250 units/mg solid, P-7000, Sigma-Aldrich, St. Louis, MO, USA) was used. A solution of pancreatin: 1.10 mg (0,1 g Pancreatin (Pancreatin, from Porcine Pancreas, P1750, Sigma-Aldrich, St Louis, MO, USA) and 0.625 g bile salts (Bile extract porcine, B8631, Sigma-Aldrich, St. Louis, MO, USA) in 25 mL of NaHCO_3_ (0.1 N) was prepared. The saliva solution was prepared mixing: 10 mL of KCl 89.6 g/L, 10 mL of KSCN 20 g/L, 10 mL of NaH_2_PO_4_ 88.8 g/L, 10 mL of NaSO4 57 g/L, 1.7 mL of NaCl 175.3 g/L, 20 mL of NaHCO_3_ 84.7 g/L, 8 mL of urea (20g/L) and was completed to 500 mL with Milli-Q water.

### 4.2. Bread Preparation

Two different baked breads were prepared in the laboratory according to the following recipe: 300 g wheat flour, 175 mL water, 20 g fresh yeast (*Saccharomyces cerevisiae*), 10 g sugar and 5 g salt. All ingredients were mixed in a commercial bread maker. The bread was baked at 200 °C for 40 min. Beetroot-enriched breads were prepared as detailed before regarding wheat flour bread but with slight modifications to obtain a bread with 20% beetroot. Beetroot dry powder was purchased from Agrosingularity S.L. (Murcia, Spain), made from dehydrated beetroot and blended.

Fortifications were made after the preparation of the bread, adding individual and mixed mycotoxins, before the digestion process, adding 1 mL of the working solutions (100 µg/mL) to 10 g of wheat bread.

### 4.3. In vitro Digestion Procedure

The procedure was the in vitro static model of Brodkorb et al. [23]. Three digestion phases were included: oral, gastric and duodenal. The assay was in triplicate for each bread, using an individually sterilized plastic bag (500 mL).

For the oral phase, 10 g of milled bread was combined for 5 min in a shaker with 6 mL of saliva solution (Section 2.1), 83 mL of milli-Q water, and 1 mL of -amylase solution. The Stomacher IUL Instrument homogenized the slurry for 30 s while simulating mastication (IUL S.A. Barcelona, Spain). An amount of 0.5 mL of pepsin solution for the stomach phase was then added to the bolus in an opaque Er-Lenmeyer flask. With HCl 6 N solution, the pH was raised to 2 to activate the stomach enzymes. The bolus was incubated for two hours at 37 °C in a darkened room while being shaken at 100 rpm (orbital shaker, Infors AG CH-4103, Bottmingen, Switzerland). After that, 1.10 mL of the pancreatin/bile salts solution was added, and the pH was then raised to 6.5 for the duodenum phase using NaHCO3 1 N (intestinal). It was shaken for two hours at 37 °C and 100 rpm. NaOH 0.5 N was used to bring the pH back to 7.2 after the incubation period.

After each stage of digestion, aliquots of each were obtained (5 or 10 mL), placed in an ice bath for 10 min to halt enzymatic activity, centrifuged for 5 min at 4500 rpm, 4 °C, and then stored until the mycotoxins and duodenal bioaccessibility were determined. The remaining duodenal phase was centrifuged in a conical flask for 10 min at 4000 rpm, 4 °C, and filtered afterwards. All collected supernatants were frozen at –20 °C. Additionally, enzyme inhibitors were utilized, It is strongly advised to use enough enzyme inhibitors to block the target digestion enzymes, as has already been demonstrated [24]. Trypsin and chymo-trypsin were both inhibited by a Bowman-Birk inhibitor (BBI; Sigma-Aldrich, cat. no. T9777). Amylase was inhibited by snap-freezing treatment and inactivation by extraction solvent and pepstatin A was employed to block pepsin (Sigma-Aldrich, cat. no. P5318). Pepstatin A was employed in a final concentration of 0.5–1.0 M in a total volume of 100 L of a BBI solution (0.05 g/L).

### 4.4. Sample Analysis

#### 4.4.1. Extraction of AFB1, OTA and ZEN from Studied Bread

Spiked bread extraction was performed according to the validated method by Juan et al. (2016) [25] with slight modifications. First, homogenized and representative portions of 2 g were weighted into 50 mL polypropylene centrifuge tubes, spiked at the studied level of digestion. After that, 10 mL of acetonitrile/water (84:16, *v*/*v*) was added and shaken using a horizontal shaking device (IKA KS 260 basic) (250 shakes/min) for 1 h. Then, tubes were centrifuged for 5 min at 4500 rpm at 5 °C with an Eppendorf Centrifuge 5810R. The supernatant was filtered on Whatman filter paper No. 4 and 5 mL of the supernatant were evaporated to dryness at 38 °C under a gentle stream of nitrogen using a multi-sample TurboVap LV Evaporator (Zymark, Hoptkinton, MA, USA). The dried extract was prepared according to the analytical determination technique used.

#### 4.4.2. Extraction of AFB_1_, OTA and ZEN from the Simulated Physiological Fluids

At each stage of the digestion process, the mycotoxins were extracted into a simulated physiological fluid. Ethyl acetate was used to do a liquid-liquid extraction in accordance with El Jai et al. ‘s instructions with a few minor adjustments [26]. The amounts of 10 mL of intestinal fluid, 5 mL of saliva, and 5 mL of stomach juice were centrifuged, with the top layer being transferred to a falcon tube. Then, using an Eppendorf centrifuge 5810R, 5 mL of ethyl acetate was added twice, agitated, and centrifuged (Eppendorf, Hamburg, Germany). Using a multi-sample TurboVap LV Evaporator, the upper layer was deposited in 15 mL PTFE centrifuge tubes and evaporated to dryness at 35 °C with a mild stream of nitrogen (Zymark, Hoptkinton, MA, USA).

#### 4.4.3. Extraction of Betalains

Betalains were analyzed in dried powdered beetroot, in prepared breads and in the simulated digestion solutions. An aliquot of dried powdered beetroot (0.1 g) or milled bread (5 g) were dissolved in 10 mL of 50% ethanol, agitated for 10 s and the homogenate centrifuged at 6000 rpm for 10 min. The simulated digestion solution (10 mL) was freeze-dried before adding the ethanol. After the centrifugation, the supernatant was collected and the same process was repeated twice to ensure maximum betalain extraction. The supernatant was evaporated to dryness using a multi-sample TurboVap LV Evaporator (Zymark, Hoptkinton, MA, USA) and redissolved with 1ml of a mixture of MeOH:H_2_O (50:50, *v*:*v*), in order to be injected in LC-Q-TOF-MS.

#### 4.4.4. Mycotoxin Analysis by LC–MS/MS

The dried residues were reconstituted to a final volume of 0.5 mL using methanol and water (70:30, *v*/*v*), and then filtered through a 13 mm/0.22 m nylon filter obtained from Analysis Vinicos S.L. (Tomelloso, Spain) before LC-MS/MS analysis. 

A 3200 QTRAP^®^ABSCIEX, outfitted with a Turbo-VTM source (ESI) interface was attached to an LC-MS/MS system made up of an LC Agilent 1200 using a binary pump and an automated injector to carry out the analysis. At 25°C, the analytes were separated chromatographically using a reverse phase analytical column Gemini^®^NX-C18 (3 µm, 150 × 2 mm ID) and security guard cartridges Gemini-NX C18 (4 × 2 mm ID). Methanol was used as phase A of the mobile phase, along with 0.1 percent formic acid and 5 mM ammonium formate, and water was used as phase B (0.1 percent formic acid and 5 mM ammonium formate). The gradient used was as follows: equilibration for two minutes at 90 percent B, linear decrease to 20 percent of phase B in three minutes, maintenance of 20 percent of phase B for one minute, linear decrease from 20 to 10 percent of phase B in two minutes, maintenance of 10 percent of phase B for six minutes, linear decrease to 0 percent B in three minutes, maintenance of 100 percent A for one minute, and then linear increase from 0 to 50 percent B in three minutes, maintenance of initial conditions (90 percent B). In every phase, the flow rate was 0.25 mL/min. Runtime was 21 min in total. The injection had a 20 L volume.

The QTRAP system was employed as a triple quadrupole mass spectrometry detector (MS/MS) for mycotoxin analysis. The Turbo-VTM source was utilized in positive mode with the following source/gas parameter settings to investigate AFs, OTA and ZEN: 3.1 vacuum gauge (10 × 10^−5^ Torr), 20 curtain gas (CUR), 5500 ion spray voltage (IS), 450 °C source temperature, and 50 each of the ion source gases (GS1 and GS2). Table 1 displays the collision energy (CE), product ions (Q3), and precursor ions (Q1). For all analytes, the entrance potential (EP) was 10 V. Data collection and processing were carried out with the use of the Analyst^®^197 program, 1.5.2. The monitored fragments (retention duration, quantification ion, and confirmation ion), as well as the spectrometric parameters (declustering potential, collision energy, and cell exit potential), were carried out earlier by Juan et al. [27].

#### 4.4.5. Betalains Analysis by LC-Q-TOF-MS

**Figure 5:**
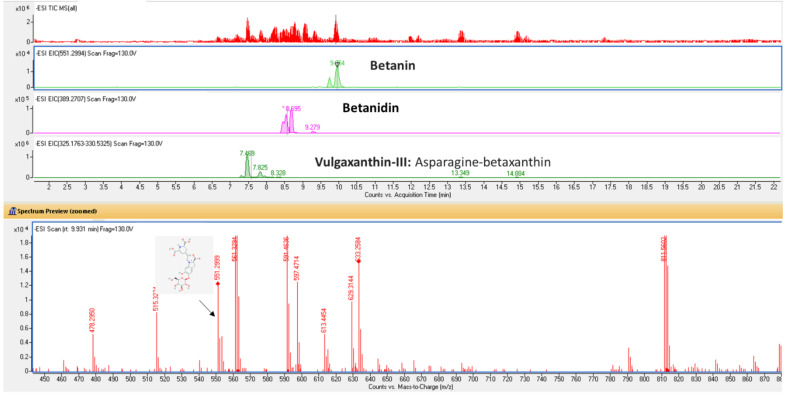
Total ion chromatograms (TIC) and extracted ion chromatograms (EIC) of betanin, betanidin and vulgaxanthin-III from red beetroot powder obtained with LC-Q-TOF-MS in negative electrospray ionization (ESI). Spectrum scan at elution time of betanin (9.931 min).

Liquid chromatography with time-of-flight mass spectrometry (LC-Q-TOF-MS) analysis was carried out using an Agilent Technologies 1200 Infinity Series LC in conjunction with an Agilent Technologies 6540 UHD Accurate-Mass LC-Q-TOF-MS (Agilent Technologies, Santa Clara, CA, USA), equipped with an electrospray ionization ion source Agilent Technologies Dual Jet Stream (Dual AJS ESI). Chromatographic separation was performed with an Agilent InfinityLab Poroshell 120 EC-C18 (3 × 100 mm, 2.7 µm) column. The mobile phase consisted of 0.1% formic acid in milli-Q water (solvent A) and methanol (solvent B). The steps of the mobile phase gradient were applied as follows: 0-2 min, maintain 10% B; 2–5 min, get 70% B; 5–7 min, get 80% B; 7–8 min, get 90% B; kept 4 min at 90% B; 12–16 min, get 95% B; 16–18 min, get 50% B; 18–22 min, return to initial conditions 10% B; the total gradient run time was 25 min. The injection volume was 10 µL, obtaining the following chromatogram of the three studied betalains in red beetroot powder (Figure 5).

The N2 drying gas flow rate 12.0 L/min, the nebulizer pressure 45 psi, capillary voltage, 3500 V; frag-mentor voltage, 130 V; skimmer voltage 65 V and octopole RF peak, 750 V, and the gas drying temperature 370 °C gas drying temperature were the Q-TOF-MS conditions. Negative ions in the range of 100–1100 m/z for MS scans and 50–600 m/z for auto MS/MS scans were obtained using a dual AJS ESI interface in negative ionization mode at scan rates of 5 s/s for MS and 3 s/s for MS/MS, respectively. The collision energy levels used for MS/MS were 20 eV, 30 eV, and 40 eV in automated mode of acquisition. In order to enable internal mass correction, two reference masses at 121.0509 and 922.0098 m/z were used. Agilent MassHunter Workstation software B.08.00 was used for instrument control and data collecting. All the MS and MS/MS data of the validation standards were integrated by MassHunter Quantitative Analysis B.10.0 (Agilent Technologies).

The main tools used for tentative identification of betalains were the interpretation of the observed MS/MS spectra (accurate mass, molecular formula and MS/MS fragmentation) in comparison with those found in the literature [28,29,30] and also by comparing their chromatographic and mass spectra characteristics with several online databases (Phenol-Explorer (Rothwell et al., 2012) [31], ChemSpider, MassBank, Spectral Database for Organic Compounds), and mass spectral data generated by authentic standards or related structural compounds (Table 2).

### 4.5. Statistical Analysis

The statistical software program Microsoft 365 Excel 2015 was used to perform the statistical analysis of the data (correlation analysis, multiple linear regression analysis, and Student’s t-test). Data from three separate experiments were expressed as (mean ± sd). The Student’s t-test for paired samples was used to statistically analyze the findings. ANOVA and the Tukey HSD post hoc test for multiple comparisons were used to statistically assess differences from the control group; *p* ≤ 0.05 was regarded as statistically significant.

## Figures and Tables

**Table 1:** Precursor ions (Q1), product ions (Q3), and collision energies (CE) used for the identification and quantification of the studied mycotoxins in bread samples.

Mycotoxin	Rt (min)	Quantitative Transition			Qualitative Transition		
		Q1 (m/z)	Q3 (m/z)	CE (eV)	Q1 (m/z)	Q3 (m/z)	CE (eV)
AFB_1_	7.4	313	241	41	313	284	39
OTA	8.5	404	239	97	404	358	27
ZEN	8.5	319	301	10	319	282	10

**Table 2:** Betalains tentatively identified by LC-Q-TOF-MS in red beet dried powder.

Betalains	Chemical Structure and Molecular Formula	RT (min)	Mass (Da)		ms/ms ^a^ [22]	Mass Error (ppm)
			Theorical	Observed		
Betanidin	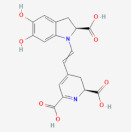 C_18_H_16_N_2_O_8_	8.6	390.2552	390.2547	345	1.28
Betanin	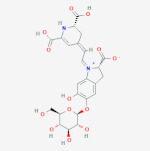 C_24_H_26_N_2_O_13_	9.9	552.3083	552.3077	389	1.08
Vulgaxanthin-III	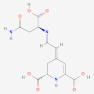 C_13_H_15_N_3_O_7_	7.3	328.2263	328.2258	277	1.52

^a^ Identification with MS fragmentation of the standard (betanin) and database (betanidin and vulgaxanthin-III) [22].

## Data Availability

Not applicable.

## References

[1] Castells M., Marin S., Sanchis A., Ramos V.J. (2008). Distribution of fumonisins and aflatoxins in corn fractions during industrial cornflake processing. J. Food Microbiol..

[2] EFSA (2012). Effect on Dietary Exposure of an Increase in Total Aflatoxin Levels from 4 µg/kg to 10 µg/kg for Dried Figs.

[3] IARC (1993). Some naturally occurring substances: Food items and constituents, heterocyclic aromatic amines and mycotoxins. IARC Monographs on the Evaluation of the Carcinogenic Risk of Chemicals to Humans.

[4] Beltrán Y., Ibánez M., Sancho J.V., Hernández F. (2009). Determination of mycotoxins in different food commodities by ultra-high-pressure liquid chromatography coupled to triple quadrupole mass spectrometry. Rapid Commun. Mass Spectrom..

[5] Copetti M., Iamanaka T., Pereira J.L., Lemes D., Nakano F., Taniwaki M. (2012). Co-occurrence of ochratoxin-A and aflatoxins in chocolate marketed in Brazil. Food Control.

[6] European Commission (2006). Regulation (EC) No 1881/2006 of 19 December 2006 setting maximum levels for certain contaminants in foodstuffs. Off. J. Eur. Union.

[7] González-Arias C.A., Piquer-García I., Marín S., Sanchís V., Ramos A.J. (2014). Bioaccessibility of ochratoxin A from red wine in an in vitro dynamic gastrointestinal model. World Mycotoxin J..

[8] Versantvoort C.H.M., Van de Kamp E., Rompelberg C.J.M. (2004). Development and Applicability of an in vitro Digestion Model in Assessing the Bioaccessibility of Contaminants from Food. RIVM Report 320102002/2004. http://www.rivm.nl/bibliotheek/rapporten/320102002.pdf.

[9] Fu Y., Shi J., Xie S.-Y., Zhang T.-Y., Soladoye O.P., Aluko R.E. (2020). Red Beetroot Betalains: Perspectives on Extraction, Processing, and Potential Health Benefits. J. Agric. Food Chem..

[10] Penalva-Olcina R., Juan C., Fernández-Franzón M., Juan-García A. (2022). Effectiveness of beetroot extract in SH-SY5Y neuronal cell protection against Fumonisin B1, Ochratoxin A and its combination. Food Chem. Tox..

[11] Morrison D.M., Ledoux D.R., Chester L.F.B., Samuels C.A.N. (2019). Occurrence of aflatoxins in rice and in cassava (Manihot esculenta) products (meal, bread) produced in Guyana. Mycotoxin Res..

[12] Zinedine A., Mañes J. (2008). Occurrence and legislation of mycotoxins in food and feed from Morocco. Food Control.

[13] Paíga P., Morais S., Oliva-Teles T., Correia M., Delerue-Matos C., Duarte S.C., Pena A., Matos Lino C. (2012). Extraction of ochratoxin A in bread samples by the QuEChERS methodology. Food Chem..

[14] Paíga P., Morais S., Oliva-Teles T., Correia M., Delerue-Matos C., Sousa A.M.M., Gonçalves M.P., Duarte S.C., Pena A., Matos Lino C. (2013). Determination of Ochratoxin A in Bread: Evaluation of Microwave-Assisted Extraction Using an Orthogonal Composite Design Coupled with Response Surface Methodology. Food Bioprocess Technol..

[15] Kulahi A., Kabak B. (2020). A preliminary assessment of dietary exposure of ochratoxin A in Central Anatolia Region, Turkey. Mycotoxin Res..

[16] Iqbal S.Z., Asi M.R., Jinap S., Rashid U. (2014). Detection of aflatoxins and zearalenone contamination in wheat derived products. Food Control.

[17] Vidal A., Morales H., Sanchis V., Ramos A.J., Marín S. (2014). Stability of DON and OTA during the breadmaking process and determination of process and performance criteria. Food Control.

[18] Keller Bol E., Araujo L., Fonseca Veras F., Welke J.E. (2016). Estimated exposure to zearalenone, ochratoxin A and aflatoxin B1 through the consume of bakery products and pasta considering effects of food processing. Food Chem. Toxicol..

[19] Slatnar A., Stampar F., Veberic R., Jakopic J. (2015). HPLC-MSn Identification of Betalain Profileof Different Beetroot (Beta vulgaris L.ssp. vulgaris) Parts and Cultivars. J. Food Sci..

[20] Sawicki T., Baczek N., Wiczkowski W. (2016). Betalain profile, content and antioxidant capacity of red beetroot dependent on the genotype and root part. J. Funct. Foods.

[21] Sobral M.M.C., Gonçalves T., Martins Z.E., Bäuerl C., Cortés-Macías E., Collado M.C., Ferreira I.M. (2022). Mycotoxin Interactions along the Gastrointestinal Tract: In Vitro Semi-Dynamic Digestion and Static Colonic Fermentation of a Contaminated Meal. Toxins.

[22] Sawicki T., Topolska J., Romaszko E., Wiczkowski W. (2018). Profile and Content of Betalains in Plasma and Urine of Volunteers after Long-Term Exposure to Fermented Red Beet Juice. J. Agric. Food Chem..

[23] Brodkorb A., Egger L., Alminger M., Alvito P., Assunção R., Ballance S., Bohn T., Bourlieu-Lacanal C., Boutrou R., Carrière F. (2019). INFOGEST static in vitro simulation of gastrointestinal food digestion. Nat. Protoc..

[24] Llorens P., Pietrzak-Fie´cko R., Moltó J.C., Mañes J., Juan C. (2022). Development of an Extraction Method of Aflatoxins and Ochratoxin A from Oral, Gastric and Intestinal Phases of Digested Bread by In Vitro Model. Toxins.

[25] Juan C., Covarelli L., Beccari G., Colasante V., Mañes J. (2016). Simultaneous analysis of twenty-six mycotoxins in durum wheat grain from Italy. Food Control..

[26] El Jai A., Juan C., Juan-García A., Mañes J., Zinedine A. (2021). Multi-mycotoxin contamination of green tea infusion and dietary exposure assessment in Moroccan population. Food Res. Int..

[27] Juan C., Oueslati S., Mañes J., Berrada H. (2019). Multimycotoxin Determination in Tunisian Farm Animal Feed. J. Food Sci..

[28] Cai Y., Sun M., Corke H. (2005). HPLC characterization of betalains from plants in the Amaranthaceae. J. Chromatogr. Sci..

[29] Li H., Deng Z., Liu R., Zhu H., Draves J., Marcone M., Tsao R. (2015). Characterization of phenolics, betacyanins and antioxidant activities of the seed, leaf, sprout, flower and stalk extracts of three *Amaranthus* species. J. Food Compos. Anal..

[30] Deladino L., Alvarez I., De Ancos B., Sánchez-Moreno C., Molina-García A.D., Schneider Teixeira A. (2017). Betalains and phenolic compounds of leaves and stems of Alternanthera brasiliana and Alternanthera tenella. Food Res. Int..

[31] Rothwell J.A., Urpi-Sarda M., Boto-Ordonez M., Knox C., Llorach R., Eisner R., Manach C. (2012). Phenol-Explorer 2.0: A major update of the Phenol-Explorer database integrating data on polyphenol metabolism and pharmacokinetics in humans and experimental animals. Database.

